# SMBFT: A Modified Fuzzy *c*-Means Algorithm for Superpixel Generation

**DOI:** 10.1155/2021/1053242

**Published:** 2021-10-08

**Authors:** Zhen Yu, Cuihuan Tian, Shiyong Ji, Benzheng Wei, Yilong Yin

**Affiliations:** ^1^Shandong Rural Credit Union, Jinan 250014, China; ^2^Health Management Center, QiLu Hospital of Shandong University, and School of Medicine, Shandong University, Jinan 250012, China; ^3^Twenty-two Institute of China Electronics Technology Group Corporation, Qingdao 266107, China; ^4^College of Science and Technology, Shandong University of Traditional Chinese Medicine, Jinan 250355, China; ^5^School of Software Engineering, Shandong University, Jinan 250101, China

## Abstract

Most traditional superpixel segmentation methods used binary logic to generate superpixels for natural images. When these methods are used for images with significantly fuzzy characteristics, the boundary pixels sometimes cannot be correctly classified. In order to solve this problem, this paper proposes a Superpixel Method Based on Fuzzy Theory (SMBFT), which uses fuzzy theory as a guide and traditional fuzzy *c*-means clustering algorithm as a baseline. This method can make full use of the advantage of the fuzzy clustering algorithm in dealing with the images with the fuzzy characteristics. Boundary pixels which have higher uncertainties can be correctly classified with maximum probability. The superpixel has homogeneous pixels. Meanwhile, the paper also uses the surrounding neighborhood pixels to constrain the spatial information, which effectively alleviates the negative effects of noise. The paper tests on the images from Berkeley database and brain MR images from the Brain web. In addition, this paper proposes a comprehensive criterion to measure the weights of two kinds of criterions in choosing superpixel methods for color images. An evaluation criterion for medical image data sets employs the internal entropy of superpixels which is inspired by the concept of entropy in the information theory. The experimental results show that this method has superiorities than traditional methods both on natural images and medical images.

## 1. Introduction

The definition of superpixel segmentation is that aggregating some pixels together to form the atomic area with certain perceptual significance for replacing the area grid. It can make use of spatial constraint information to be robust to certain noises [1]. Meanwhile, superpixel is an adaptive region, so its feature-based statistical information is superior to features based on the fixed regular region in artificial segmentation.

Superpixel can not only strengthen the local consistency of the image but also keep the original boundary information of the image. The middle level features of the image can be extracted in the atomic area segmented by the superpixel method, which is beneficial for further processing of the image. Compared with the pixel-level operation, the superpixel method has some advantages such as lower time complexity [2]. Besides, the superpixel method is more robust to intensity inhomogeneity. Some images have the intensity inhomogeneity problem; for example, gray value of white matter in some areas of brain MR image is close to that of gray matter in other areas and even is lower than that of gray matter [3]. As in the atomic area, the contrast ratio of the internal gray values in one superpixel is higher, and there is no intensity inhomogeneity phenomenon in the superpixel, which can avoid the influence of intensity inhomogeneity [4].

This paper proposes a new superpixel segmentation method. It first starts with an initial rough clustering, and then a new fuzzy theory-based objective function is used as the target function, to obtain more accurate segmentation results. This process will be repeated until convergence. The method can make full use of the advantages of fuzzy clustering methods and alleviate the disadvantages of rigid segmentation of traditional superpixel segmentation methods.

The paper consists of five sections.  Section 1 is the introduction, which briefly introduces the basic knowledge of superpixel and analyzes problems of the traditional superpixel methods.  Section 2 mainly reviews related methods.  Section 3 begins with the introduction of the existing problems in the current work, which in turn leads to the motivation of the proposed superpixel algorithm. The proposed algorithm is evaluated on the natural images and medical images with strong fuzziness in Section 4.  Section 5 concludes and gives the future work.

## 2. Related Works

Recently, superpixel segmentation methods are becoming more and more popular. These methods can be mainly divided into two categories: graph-based methods and gradient ascent methods [5–12].

The basic idea of the superpixel method is to use a weighted undirected graph to represent an image. Each pixel in the image corresponds to one node on the net chart, and the relationship between pixel and pixel corresponds to the edge of undirected graph. And the similarity between pixel and pixel is obtained by calculating the weight of edges between nodes. The nodes on the undirected graph are segmented based on some segmentation rules to generate superpixels. This is a kind of top-down segmentation methods. Its typical algorithms include Graph-based segmentation [13], Normalized cuts [14], Superpixel lattice [15], GCa10 and GCb10 [16], Entropy Rate Superpixel Segmentation [17], and Superpixels via Pseudo-Boolean Optimization [18].

The Graph-based segmentation is based on a minimum spanning tree, with the goal of making pixels in the same area consistent. Minimum spanning tree is a superpixel which is obtained by clustering nodes on the graph. These methods are fast, but they cannot control the accuracy and number of superpixels [5]. The Normalized cuts are used for segmenting subgraph and using certain criterion to measure the segmentation result. Its strategy is to find a kind of segmentation method to minimize the cost function between subgraphs. The advantage of Normalized cuts algorithm is that the number of superpixel can be artificially defined, the shape is relatively compact, and the area of each superpixel is relatively closed. But the speed of generating superpixel with the Normalized cuts algorithm is slow, so the running time will be longer for larger images. Such algorithm is suitable for the skeleton extraction, model estimation, etc. The Superpixel lattice is inputting boundary graph of the image, searching the minimum weight path passing through the image, and making the segmentation in the smallest space of the boundary cost graph. It is a greedy algorithm which can keep the topological structure of the image. In the entropy rate superpixel, an energy function is designed based on graph topology. The function has two parts: one is image random walk entropy rate which is used to form the compact and even cluster for keeping internal media of superpixel, and the other is used to balance the items which are used to make the cluster similar and size-uniform.

The segmentation methods based on gradient ascent start with an initial rough clustering. In this process, gradient ascent is used to continuously improve the result of the previous iteration, to obtain better segmentation results. This process will be repeated until convergence. Representative algorithms include Watershed [19], Mean Shift [20], Quick Shift [21], Turbo pixel [22], SLICO [5], and SEEDS [23].

The Watershed method describes the image as a topological topographic map, with gray level of pixel representing altitude of this point. The so-called watershed refers to the boundary of each reception basin, and reception basin refers to the local minimum value and area under its influence. This algorithm is simple, has low computing complexity, and can accurately locate the target. Its disadvantage is that it may cause serious oversegmentation. The Mean Shift method is the quick statistical iteration algorithm. It has no parameters and is estimated based on kernel density gradient. It calculates the vector of Mean Shift of all feature space data points in the kernel window and then moves towards the direction of gradient ascent until reaching the maximum convergence density. This method has better stability and noise immunity but lacks image semantic information during the segmentation. A Quick Shift method is similar to the Mean Shift segmentation algorithm, which is also a kind of segmentation method based on the gradient ascent method. It segments the image by continuously promoting the move of each data point in the pixel feature space, and the specific moving direction is the nearest pixel direction which can increase density estimation of Parzen [21]. This algorithm does not need loop, but the shape and number of superpixel cannot be artificially controlled, and the compact degree between superpixels is not very ideal. So, it is precisely suitable for the field of image segmentation without high requirements for compact degree, such as target positioning and motion segmentation. The Turbo pixel makes random selection of a certain number of seed points from the image, uses level set for expansion, and controls the generation of superpixel by curvature evolution model and skeletonization process of background region. The number of superpixel obtained by Turbo pixel is controllable, size is relatively uniform, boundary is relatively close to the actual boundary of the image, and this method has rapid processing speed. The SLICO method is a simple and convenient superpixel algorithm. It obtains the similarity between pixels by calculating the color information and spatial information between pixel and pixel and then making clustering of pixels. This algorithm can effectively generate the compact superpixel with high degree of homogenization. For the SLICO algorithm, only the number of superpixel is needed. Its running time and storage space are linear, the generated superpixel is compact and relatively ideal, size is consistent, and shape is uniform. With obvious advantage, the SLICO algorithm is widely applied to the current image processing algorithms.

## 3. Superpixel Method Based on Fuzzy Theory

### 3.1. Problem

The current study on superpixel has made a lot of fruitful results and also has been used in various fields, such as mitochondrial segmentation of electron micrograph, human pose estimation, the moving target tracking. Superpixels generated by Normalized cuts, Turbo pixel, SLICO, and other algorithms all have a compact structure and uniform shape, but a compact structure makes the superpixel fail to cover the whole information of a target accurately, and a uniform shape makes the target at different scales have different semantic levels during the superpixelation.

**Figure 1:**
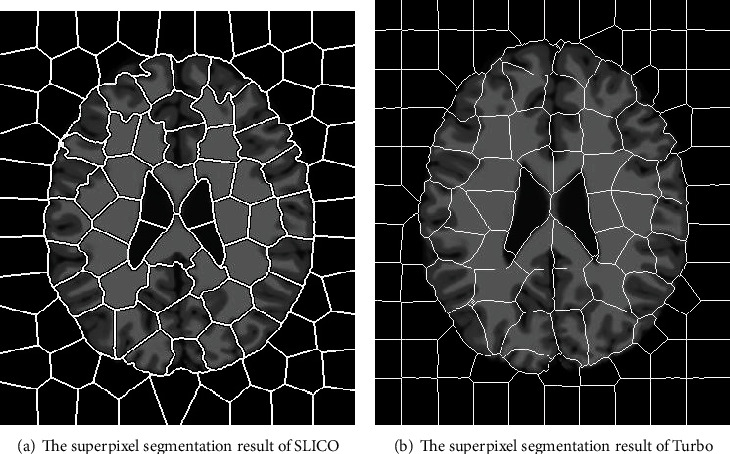
Superpixel segmentation of brain image in a traditional method.

In addition, it can be seen from Figure 1 intuitively that when the latest superpixel method is used to make the segmentation of brain MR image with stronger fuzziness, most superpixels contain various brain tissues (including white matter, gray matter, and cerebrospinal fluid). In principle, one superpixel should contain the single medium; otherwise, much inconvenience will be brought to the processing of such image. The reason is that the traditional superpixel algorithm is aimed at the natural images, and classification of boundary pixel points basically adopts a two-valued logic segmentation method. However, when these methods are used to process the image with significant fuzziness, the boundary between different tissues cannot be classified correctly, so the segmentation is not ideal. Generated superpixels often contain multiple target tissues at the same time, which negatively affects following image processing.

In order to segment the boundary between different tissues more correctly during the processing of blurred image, the paper proposes a Superpixel Method Based on Fuzzy Theory by combining the advantages of fuzzy theory in describing uncertainty elements. The algorithm in the paper takes the fuzzy theory knowledge as the guide and the traditional fuzzy *c*-means clustering algorithm as the basis. And considering that the generated superpixel cannot produce cross, it deals with such situation by adding the coordinate distance of pixel point; designs the objective function, to obtain the approximate optimal solution expression with the Lagrangian multiplier method; and finally gets the superpixel segmentation result by unceasing iterative optimization. Meanwhile, considering the factor that the brain image is easily affected by the noise due to imaging device, this paper employs neighborhood information for denoising, which effectively overcomes the influence of noise and makes the algorithm proposed in the paper be robust. The algorithm in the paper is applied to the natural images in the Berkeley database and brain MR images with relatively obvious fuzziness in Brain web. The detailed experimental results will be presented in Section 4.

### 3.2. Algorithm Description

The method proposed in this paper is to design a new objective function on the basis of fuzzy *c*-means. Its key points lie in the following: (1) in addition to gray level or color distance in the distance formula, the coordinate distance of pixel point is also added to control the weight of pixel classification, avoiding the cross of superpixel, and (2) neighborhood information is added to eliminate noise interference.

**Figure 2:**
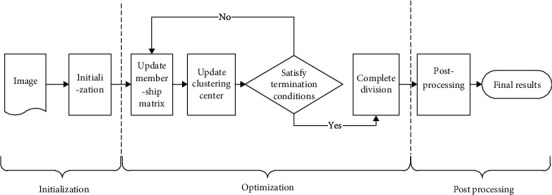
Flow chart of the proposed method.

It mainly includes the following steps: (1) make initialization operation of the image, and set the specific number of pixels; (2) conduct optimization solution of objective function with the Lagrange multiplier method to get the approximate optimal solution of membership matrix equivalent variation; and (3) conduct the reprocessing according to the result of step (2) to get the final result. The specific process is shown in Figure 2.

### 3.3. Initialization

First of all, we segment the image into the uniform and regular grid according to the number of superpixels. For the image with size of *M* × *N*, 0 ≤ *x* ≤ *M*, and 0 ≤ *y* ≤ *N*, *Z*(*x*, *y*) represents the whole image grid. The segmentation is to segment *Z* into *n* nonnull subregions *z*_1_, *z*_2_, ⋯, *z*_*n*_ meeting the following five conditions.

1. ∪_*i*=1_^*n*^*z*_*i*_ = *Z*
2. For all *i*, *j*, when *i* ≠ *j*, *z*_*i*_∩*z*_*j*_ = ∅
3. For all *z*_*i*_,  *i* = 1, 2, ⋯, *n*, *P*(*z*_*i*_) = true
4. For all *i*, *j*, when *i* ≠ *j*, *P*(*z*_*i*_∩*z*_*j*_) = false
5. For all *z*_*i*_,  *i* = 1, 2, ⋯, *n*, *z*_*i*_ are the connected region

In the above conditions, *P*(*z*_*i*_) is the logic predicate of elements in all subregions *z*_*i*_ (*i* = 1, 2, ⋯, *n*), with ∅ representing null set. The side length of each grid is $S=\sqrt{M\times N/k}$, and *M* × *N* represents the set of all pixels. The central point of each grid is selected as the initial clustering center, and in order to avoid the noise interference, random disturbance should be made here, i.e., selecting 3 × 3 of the template around the initial clustering center and taking the point with smallest gradient as a new clustering center.

### 3.4. Objective Function Design

The traditional fuzzy *c*-means clustering (FCM) method performs the image segmentation by clustering of pixel points and has a good segmentation effect on the image with stronger fuzziness [24, 25]. However, the traditional FCM method cannot directly generate superpixel. The method proposed in this paper adds the coordinate distance for judgment besides judging categorical attributes with gray level distance on the basis of the traditional FCM. The function of coordinate distance lies in generating the compact category area and avoiding cross between different categories. In addition, to enhance the robustness of the algorithm, the paper uses the neighborhood information in the objective function to reduce the impact of noise on superpixel segmentation [26].

Objective function designed in the paper is

(1) $$\begin{array}{c} J_{m}=\overset{c}{\underset{i=1}{\sum }}\overset{N}{\underset{k=1}{\sum }}u_{ik}^{p}{\left\|y_{k}-v_{i}\right\|}^{2}+\alpha \overset{c}{\underset{i=1}{\sum }}\overset{N}{\underset{k=1}{\sum }}u_{ik}^{p}\left({\left.\;|\;X_{k}-X_{i}\right\|}^{2}+{\left\|Y_{k}-Y_{i}\right\|}^{2}\right)+\frac{\beta }{N_{R}}\overset{c}{\underset{i=1}{\sum }}\overset{N}{\underset{k=1}{\sum }}u_{ik}^{p}\underset{{\text{y}}_{r}\in N_{k}}{\sum }\;|\;{\left\|y_{\text{r}}-v_{i}\right\|}^{2}. \end{array}$$

The formula can be divided into three parts. The first part is the objective function of the traditional fuzzy *c*-means clustering algorithm, where {*y*_*k*_, *k* = 1, 2, ⋯, *N*} represents the set of gray value of the image, *c* represents the predetermined number of categories, {*v*_*i*_, *i* = 1, 2, ⋯, *c*} represents each clustering center, *p* represents the index of membership function for controlling the fuzziness of clustering results, and ‖*y*_*k*_ − *v*_*i*_‖^2^ represents the distance between pixel point and clustering center. The second part is the information about the coordinate distance of pixel points, where *X* and *Y*, respectively, represents the horizontal and vertical coordinates and *α* is used to control the weight of coordinate distance of the entire distance. Parameter *α* needs to be set according to the number of superpixel, with an approximate range of 1-10; the larger the number, the smaller the value. The function of this part is to generate superpixel and avoid the cross between category and category. The third part is about the neighborhood information of pixel point, where *N*_*k*_ represents the surrounding neighborhood pixel of the current pixel point, *N*_*R*_ is the base number of *N*_*k*_ and set as 8 in this paper, representing 8 as the neighborhood of pixel point, *β* is used to control the weight of neighborhood information of the overall information. Parameter *β* is set according to the specific experimental results. The value used in this experiment is 1. The significance of this part lies in that the classified discrimination of pixel point also considers categorical information of surrounding neighborhood pixels and avoids the impact of noise inside the image on the pixel classification.

In addition, the objective function contains an implicit constraint condition; i.e., the sum of membership degree of each pixel is 1. The formula is as follows:

(2) $$\begin{array}{c} g=1-\overset{c}{\underset{i=1}{\sum }}u_{ik}. \end{array}$$

Objective function with constraint condition can be obtained by formulas (1) and (2):

(3) $$\begin{array}{c} F_{m}=J_{m}+\lambda g. \end{array}$$

That is *F*_*m*_ = ∑_*i*=1_^*c*^∑_*k*=1_^*N*^(*u*_*ik*_^*p*^*D*_*ik*_ + (*β*/*N*_*R*_)*u*_*ik*_^*p*^*γ*_*i*_) + *λ*(1 − ∑_*i*=1_^*c*^*u*_*ik*_); i.e., *D*_*ik*_ and *γ*_*i*_, respectively, are

(4) $$\begin{array}{c} D_{ik}=\left({\left\|y_{k}-v_{i}\right\|}^{2}+\alpha {\left\|X_{k}-X_{i}\right\|}^{2}+\alpha {\left\|Y_{k}-Y_{i}\right\|}^{2}\right),\gamma_{i}=\left(\underset{{\text{y}}_{r}\in N_{k}}{\sum }{\left\|y_{\text{r}}-v_{i}\right\|}^{2}\right). \end{array}$$

The Lagrange multiplier method is a kind of optimization algorithm under the equality constraint condition, so we can use such method to solve the optimal solution expression form of variables in the objective function.

Use the above formula to seek partial derivative of *u*_*ik*_ to obtain

(5) $$\begin{array}{c} \frac{\partial F_{m}}{\partial u_{ik}}={pu}_{ik}^{p-1}D_{ik}+\frac{p\beta }{N_{R}}u_{ik}^{p-1}\gamma_{i}-\lambda . \end{array}$$

Saying the above formula is equal to 0 to obtain *u*_*ik*_^∗^,

(6) $$\begin{array}{c} u_{ik}^{*}={\left(\frac{\lambda }{p\left(D_{ik}+\left(\beta /N_{R}\right)\gamma_{i}\right)}\right)}^{1/\left(p-1\right)}. \end{array}$$

Due to ∑_*j*=1_^*c*^*u*_*jk*_ = 1, for any *k*, we can obtain

(7) $$\begin{array}{c} \overset{c}{\underset{j=1}{\sum }}{\left(\frac{\lambda }{p\left(D_{jk}+\left(\beta /N_{R}\right)\gamma_{j}\right)}\right)}^{1/\left(p-1\right)}=1. \end{array}$$

Further obtain:

(8) $$\begin{array}{c} \lambda =\frac{p}{{\left(\sum_{j=1}^{c}{\left(1/p\left(D_{jk}+\left(\beta /N_{R}\right)\gamma_{j}\right)\right)}^{1/\left(p-1\right)}\right)}^{\left(p-1\right)}}. \end{array}$$

Substitute *λ* into *u*_*ik*_^∗^ to obtain

(9) $$\begin{array}{c} u_{ik}^{*}=\frac{1}{\sum_{j=1}^{c}{\left(\left(D_{ik}+\left(\beta /N_{R}\right)\gamma_{i}\right)/\left(D_{jk}+\left(\beta /N_{R}\right)\gamma_{j}\right)\right)}^{1/\left(p-1\right)}}. \end{array}$$

Use function to seek partial derivative of *v*_*i*_ to obtain

(10) $$\begin{array}{c} \frac{\partial F_{m}}{\partial v_{i}}=\overset{N}{\underset{k=1}{\sum }}u_{ik}^{p}\left(y_{k}-v_{i}\right)+\overset{N}{\underset{k=1}{\sum }}u_{ik}^{p}\frac{\beta }{N_{R}}\underset{{\text{y}}_{r}\in N_{k}}{\sum }\left(y_{\text{r}}-v_{i}\right). \end{array}$$

Saying the above formula is equal to 0 to obtain *v*_*i*_^∗^,

(11) $$\begin{array}{c} v_{i}^{*}=\frac{\sum_{k=1}^{N}u_{ik}^{p}\left(y_{k}+\left(\beta /N_{R}\right)\sum_{y_{r}\in N_{k}}y_{r}\right)}{\left(1+\beta \right)\sum_{k=1}^{N}u_{ik}^{p}}. \end{array}$$

Use the function to seek partial derivative of *X*_*i*_ and *Y*_*i*_ to obtain

(12) $$\begin{array}{c} \frac{\partial F_{m}}{\partial X_{i}}=\overset{c}{\underset{i=1}{\sum }}\overset{N}{\underset{k=1}{\sum }}u_{ik}^{p}*2\left(X_{k}-X_{i}\right),\\ \frac{\partial F_{m}}{\partial Y_{i}}=\overset{c}{\underset{i=1}{\sum }}\overset{N}{\underset{k=1}{\sum }}u_{ik}^{p}*2\left(Y_{k}-Y_{i}\right). \end{array}$$

Saying the above two formulas are equal to 0 to obtain *X*_*i*_^∗^ and *Y*_*i*_^∗^,

(13) $$\begin{array}{c} X_{i}^{*}=\frac{\sum_{k=1}^{N}u_{ik}^{p}X_{k}}{\sum_{k=1}^{N}u_{ik}^{p}}, \end{array}$$

(14) $$\begin{array}{c} Y_{i}^{*}=\frac{\sum_{k=1}^{N}u_{ik}^{p}Y_{k}}{\sum_{k=1}^{N}u_{ik}^{p}}. \end{array}$$

Equations (9), (11), (13), and (14), respectively, represent the membership matrix, clustering center gray value, optimal solution expression form of horizontal coordinate, and optimal solution expression form of vertical coordinate

Among the following steps, we conduct the iterative update of relevant variables with these expressions.

1. Calculate membership matrix with formula (9)
2. Calculate the gray value and coordinate value of clustering center with formulas (11), (13), and(14)
3. Repeat steps (1) and (2) until meeting the termination conditions. The termination condition of this method is that the change of gray value of clustering center is less than the predefined thresholds or the number of iterations is more than a predefined value set

Due to various reasons, the natural image will have fuzziness inevitably, so it is necessary to expand the algorithm in this paper to natural image. The processing of natural image is basically consistent with that of gray level image, so we only need to replace the gray level distance in the objective function with the color distance. The paper defines the distance by converting gray level distance into three color channels under Lab space. The objective function is as follows:

(15) $$\begin{array}{c} J=\overset{c}{\underset{i=1}{\sum }}\overset{N}{\underset{k=1}{\sum }}u_{ik}^{p}\left({\left\|{\text{l}}_{k}-v_{1i}\right\|}^{2}+{\left\|{\text{a}}_{k}-v_{2i}\right\|}^{2}+{\left\|{\text{b}}_{k}-v_{3i}\right\|}^{2}\right)+\alpha \overset{c}{\underset{i=1}{\sum }}\overset{N}{\underset{k=1}{\sum }}u_{ik}^{p}\left({\left\|X_{k}-X_{i}\right\|}^{2}+{\left\|Y_{k}-Y_{i}\right\|}^{2}\right)+\frac{\beta }{N_{R}}\overset{c}{\underset{i=1}{\sum }}\overset{N}{\underset{k=1}{\sum }}u_{ik}^{p}\underset{{\text{y}}_{r}\in N_{k}}{\sum }\left({\left\|{\text{l}}_{k}-v_{1i}\right\|}^{2}+{\left\|{\text{a}}_{k}-v_{2i}\right\|}^{2}+{\left\|{\text{b}}_{k}-v_{3i}\right\|}^{2}\right). \end{array}$$

Other steps are completely consistent with the processing method of gray level image.

### 3.5. Final Result Obtained by Postprocessing

The approximate optimal membership matrix can be obtained by the previous steps, and the category label of each pixel can be obtained with such matrixes. Pixels within the same category are taken as a superpixel, getting the segmentation result.

However, there will be some isolated points after such processing. For some isolated points, we can use the interconnection algorithm to conduct the postprocessing: based on the preliminary segmentation result, we set a threshold and calculate the number of pixels in each superpixel. If the number of pixels in one superpixel is lower than such threshold, we take it as the isolated point set, and we will find the superpixel set adjoining these isolated point sets by calculating the average gray level value and combine them together to get the final segmentation result of superpixel.

## 4. Experimental Results

### 4.1. Experimental Results of Natural Color Image Segmentation

Since natural color images may get noise pollution during the imaging due to uneven distribution of background light or vibration of sensor, etc., leading to the reduction of image quality, so a lot of information has uncertainty. Unluckily, the traditional superpixel method cannot solve this problem effectively. Owing to the proposed algorithm in this paper based on the fuzzy theory, it can overcome the impact brought by this uncertainty to some extents. Here, some experiments are performed to prove the effectiveness of the algorithm in this paper.

Like traditional superpixel methods, Berkeley database [27], a natural image data set, is used in the experiments, and evaluation criterion we used includes Undersegmentation error and Boundary recall. In addition, the traditional superpixel method does not describe the importance of the above two criteria, and one criterion may be more suitable for choosing superpixel according to different applications. Therefore, the paper proposes a new evaluation criterion: average accuracy rate. Such criterion is under the comprehensive consideration of the combination of UE with BR, and different weights can be assigned to different indicators by corresponding to different applications for better providing reference for the choice of superpixel method.

In order to further explain these evaluation indexes, *R* = {*R*_1_, *R*_2_, ⋯, *R*_*n*_} represents the segmentation result of ground truth, in which *n* represents the number of targets and ∣*R*_*i*_∣ represents the size of targets.

1. Undersegmentation error (UE), given in (16), is used to measure the matching degree of superpixel and target object. In principle, one superpixel can only belong to a target object. UE represents the proportion of the pixels disclosed inside the superpixel of the target object. Big UE value means large matching degree of the superpixel and the target object:

(16) $$\begin{array}{c} \text{UE}=\frac{\sum_{i}\sum_{k:S_{k}\cap R_{i}\neq \emptyset }\;|\;S_{k}-R_{i}\;|\;}{\sum_{i}\;|\;R_{i}\;|\;}, \end{array}$$

where *S*_*k*_ represents the number of pixel disclosed inside the superpixel of target *R*_*i*_ segmented by ground truth

**Figure 3:**
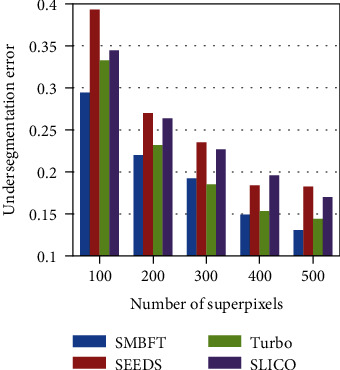
UE value histogram.

The traditional gradient ascent-based superpixel methods, including Turbo, SLICO, and SEEDS, are compared with the proposed SMBFT, and the results are given in Figure 3 and Table 1.

(2) Boundary recall (BR), given in (17), measures the accuracy of segmentation boundary. BR is the coincidence rate of superpixel boundary and real target object boundary. The higher the BR value is, the more coincidence rate the boundary of superpixel and the boundary of target object has:

(17) $$\begin{array}{c} \text{BR}=\frac{\sum_{p\in \delta R}f\left(\min_{q\in \delta S}\left\|p-q\right\|<\varepsilon \right)}{\left|\delta R\right|}, \end{array}$$

where *δS* and *δR*, respectively, represent the boundary set segmented by superpixel and the boundary set of ground truth. Function *f* is used to detect whether the difference between two boundaries is less than *ε* pixels. The value of *ε* is 2.

**Figure 4:**
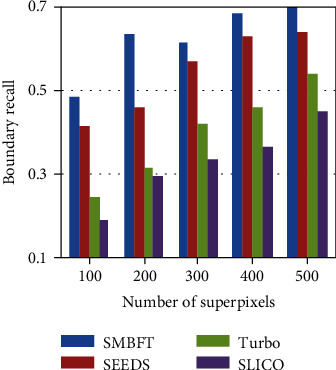
BR value histogram.

The BR values of the traditional methods and the proposed methods are listed in Figure 4 and Table 2.

(3) Average accuracy rate (AAR), given in (18), is designed in the comprehensive consideration of the first two evaluation indexes:

(18) $$\begin{array}{c} \text{AAR}=\mu \left(1-\text{UE}\right)+\left(1-\mu \right)\text{BR}, \end{array}$$

in which *μ* ∈ (0, 1) represents the weight the evaluation index and its value is 0.5. The higher the AAR value is, the better segmentation results we get

**Figure 5:**
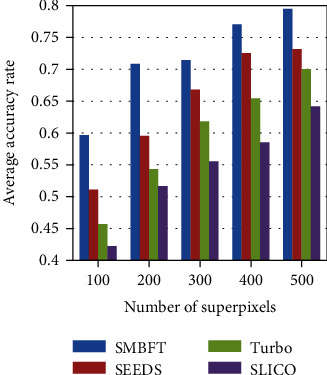
AAR value histogram.

The AAR values of the traditional methods and the proposed methods are listed in Figure 5 and Table 3.

The results of the above three evaluation criteria (i.e., UE, BR, and AAR) show that the SMBFT algorithm proposed in the paper is superior to the traditional superpixel methods. Taking the segmentation of 100 superpixel blocks as an example, the UE value of SMBFT is 4%-10% lower, the BR value is 7%-30% higher, and the AAR value is 8%-17% higher than other algorithms. In addition, all the superpixel algorithms have a common trend that three evaluation criteria tend towards the extreme with the increase of segmentation blocks of superpixel. Seeing from the extreme, if each pixel is taken as one superpixel, the UE value will be 0, and the BR and AAR value is 1.

The result also shows that for the natural images with fuzziness, the proposed fuzzy theory based superpixel method can work well.

**Figure 6:**
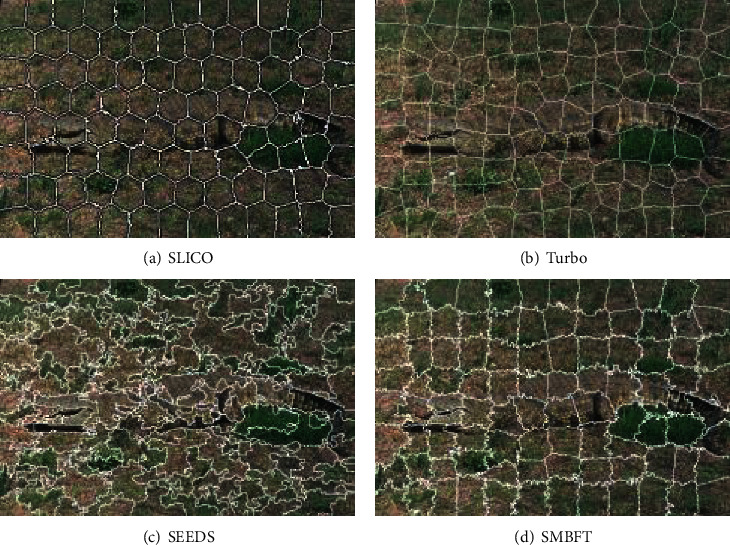
(a)–(d) represent the superpixel segmentation result of SLICO, Turbo, SEEDS, and SMBFT methods.

**Figure 7:**
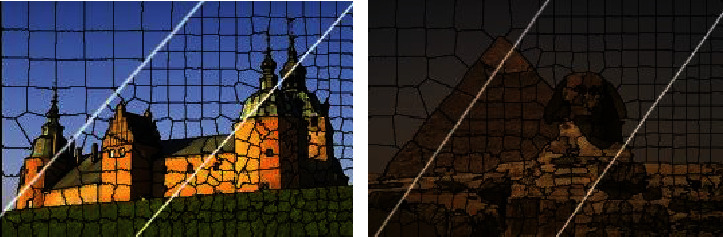
Result of segmenting 100, 300, and 500 superpixels with the SMBFT method.

In the following, the visual superpixel segmentation result of natural image is given in Figures 6 and 7.

Figures 6 and 7 show that superpixel generated by the SLICO and Turbo algorithm is uniform and regular, but the degree of matching target boundary is poor, which brings inconvenience to the further processing of the image. Although the SEEDS algorithm has good matching degree, superpixel is disordered and irregular, which is also not beneficial to the subsequent steps of image processing. The algorithm in the paper keeps good matching degree, and superpixels are relatively regular and uniform.

### 4.2. Experimental Results of Brain MR Image Segmentation

Since medical image itself is challenging, the performance of superpixel segmentation with traditional method is not ideal. The classification of boundary pixels may be not correct, and superpixels segmented contain various target tissues, which are not helpful to further image processing. In order to verify the advantage of the method in this paper in medical image with significant fuzziness, the paper selects the database with higher recognition in the field of medical image processing to do experiment, and details are as follows.

For medical image data set, we select the brain image data set artificially synthesized in brain web [28] website, and we artificially define various parameters, such as noise and bias field. Noise parameters selected in this paper are 0%, 5%, and 7%, respectively, and bias field parameters are 0% and 40%, respectively. Since the data set does not has the ground truth for calculate UE and BR, evaluation criterion adopted by the paper does not use the traditional UE and BR.

Considering the entropy value represents the quantity of information, the paper uses the average entropy value of superpixels to measure the final superpixel segmentation result. If the entropy value of superpixel is smaller, target segmentation inside the superpixel is more single, and the segmentation performance is better.

The specific calculation is defined as follows:

(19) $$\begin{array}{c} H\left(p\right)=-\underset{\left(i,j\right)}{\sum }p\left(i,j\right)\ln p\left(i,j\right), \end{array}$$

where *p*(*i*, *j*) = *x*(*i*, *j*)/∑_(*i*, *j*)_*x*(*i*, *j*), where *x*(*i*, *j*) represents image pixel.

**Figure 8:**
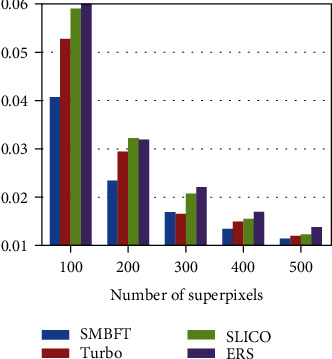
Entropy value histogram of image with noise of 0 and bias field of 0.

**Figure 9:**
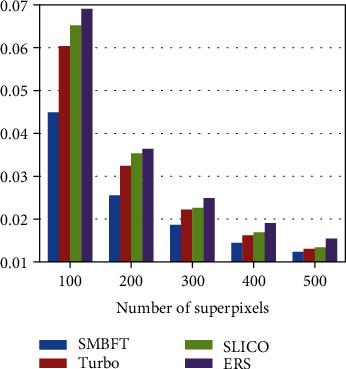
Entropy value histogram of image with noise of 5% and bias field of 40%.

**Figure 10:**
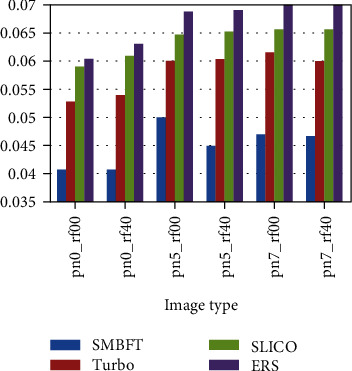
Entropy value histogram of image with 100 superpixel blocks.

**Figure 11:**
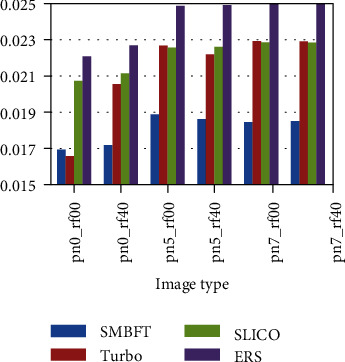
Entropy value histogram of image with 300 superpixel blocks.

**Figure 12:**
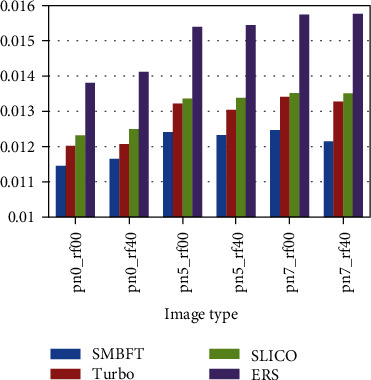
Entropy value histogram of image with 500 superpixel blocks.

The superpixel methods compared in the paper includes Turbo, SLICO, and ERS. The results are displayed in two ways: one is fixing image type, i.e., doing experiment of different segmentation blocks on the same type of image; and the other is fixing superpixel blocks, i.e., doing experiment of different image types under the same scale. Specific results are as follows:

1. Fixing image type for comparison:

(2) Fixing superpixel blocks for comparison:

The experimental result (Figures 8–12, Tables 4–8) shows that the experimental result obtained by 100-500 superpixel blocks segmented with the algorithm in this paper is far better than that obtained with the traditional superpixel algorithm, and the effect is more significant, especially in the case of less segmentation blocks. Taking 100 blocks as an example, the entropy value of the algorithm in this paper is about 2%, less than conventional methods. Although it is less intuitive than a natural image in a numerical value, yet since its entropy value is smaller, the algorithm in this paper has more obvious advantages in medical image. The reason is that fuzziness of medical image is much higher than that of common natural image due to its features, and the algorithm in this paper can deal with the fuzziness of medical image specially based on the fuzzy theory, making up the disadvantage of rigid segmentation of the traditional superpixel algorithms in processing such image. Pixel points with higher uncertainty in boundary can be classified correctly with the maximum probability, making that the matching degree of superpixel boundary generated by segmentation and original image boundary become higher, and targets inside superpixel also are single and medium is uniform. In addition, the function design also increases spatial constraint information and determines the category of current pixel by judging the category of neighborhood pixels, effectively avoiding the noise interference. Therefore, in the case of noise parameters increasing gradually, the algorithm in this paper remains the better segmentation result and more robust.

**Figure 13:**
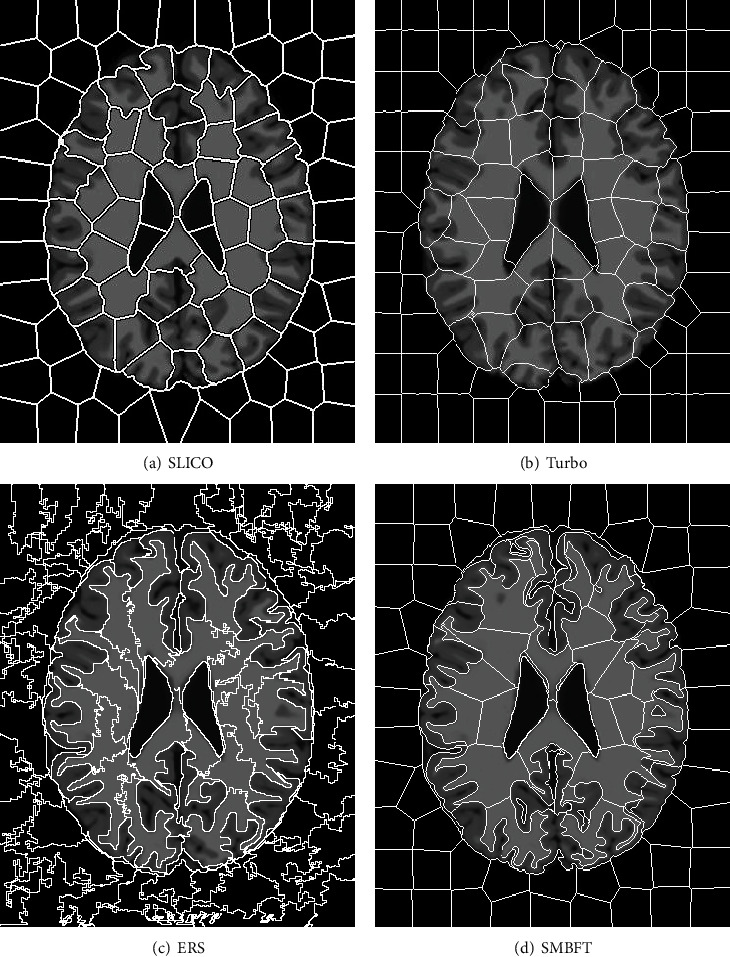
(a)–(d) represent the superpixel segmentation result of SLICO, Turbo, ERS, and SMBFT methods.

Figure 13 is the intuitive result on brain MR image with different superpixel methods.

Figure 13 shows that superpixel generated by the SLICO and Turbo algorithms is uniform and regular, but the degree of matching target boundary is poor, and many superpixels contain multiple target tissues, which brings inconvenience to the further processing of the image. Although the ERS algorithm has good matching degree, superpixel is disordered and irregular, which is also not beneficial to the subsequent steps of image processing. However, the proposed algorithm in the paper keeps good matching degree, and superpixels are relatively regular and uniform, with obvious advantage.

Above all, considering the image has the certain fuzziness, and different images have different fuzziness degrees, the proposed method in this paper uses the fuzzy clustering to design a formula and introduces the fuzzy theory into the superpixel segmentation, which can effectively solve this kind of problem. Therefore, the experimental result shows that the method in this paper is superior to the traditional superpixel methods.

## 5. Conclusion and Discussion

The algorithm in this paper makes full use of the advantages of fuzzy theory in dealing with fuzziness. Meanwhile, the paper also considers the influence of noise on image segmentation and solves it by increasing the use of surrounding neighborhood information of pixel points in the objective function, making our algorithm have better robustness. Due to the widespread existence of fuzziness on the nature images, the method also has better generalization.

In terms of application, experimental results show the effectiveness on two kinds of images. Besides, a new evaluation criterion is proposed from the perspective of how to select the superpixel under different application backgrounds for measuring different evaluation criterions and different weight problems and providing a new idea for how to select the superpixel for algorithms. In addition, the paper firstly verifies a superpixel segmentation method on challenging medical image sets. With the inspiration of the information theory, the paper first introduces the entropy value and uses it as the evaluation criterion of superpixel segmentation result, obtaining the relatively better verification results. All experimental results show that the algorithm in this paper is superior to the traditional superpixel methods, which also proves the effectiveness to use fuzzy theory to deal with the problem.

Though the proposed algorithm has achieved good segmentation results, there are still certain limitations. When the number of superpixel block required is small, our algorithm can achieve good results. But if the number is large and because our algorithm conducts the iterative processing on the basis of the global function, the time complexity is higher, and it is not suitable for batch processing. So, how to further optimize the algorithm in this paper and reduce the time complexity is a future problem. In addition, in view of different algorithms, how many superpixels should be used and the applications in different dimensions would also be worth considering.

## Figures and Tables

**Pseudocode 1:**
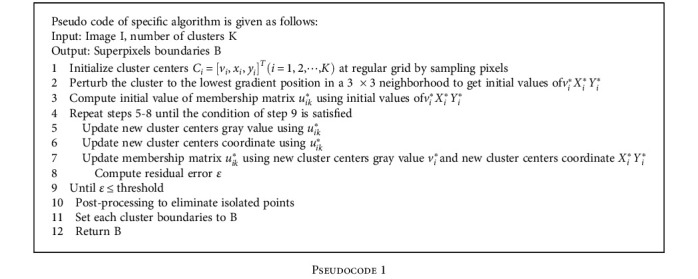


**Table 1:** UE value of several superpixel methods.

	100	200	300	400	500
SMBFT	0.2942	0.2199	0.1923	0.1493	0.1308
SEEDS	0.3932	0.2701	0.2350	0.1840	0.1823
Turbo	0.3326	0.2319	0.1851	0.1534	0.1440
SLICO	0.3445	0.2637	0.2269	0.1959	0.1701

**Table 2:** BR value of several superpixel methods.

	100	200	300	400	500
SMBFT	0.4879	0.6370	0.6208	0.6901	0.7201
SEEDS	0.4156	0.4609	0.5711	0.6350	0.6458
Turbo	0.2464	0.3187	0.4215	0.4624	0.5445
SLICO	0.1894	0.2966	0.3379	0.3665	0.4535

**Table 3:** AAR value of several superpixel methods.

	100	200	300	400	500
SMBFT	0.5968	0.7085	0.7142	0.7704	0.7947
SEEDS	0.5112	0.5954	0.6680	0.7253	0.7317
Turbo	0.4569	0.5434	0.6182	0.6545	0.7002
SLICO	0.4224	0.5165	0.5555	0.5853	0.6417

**Table 4:** Entropy value of image with noise of 0 and bias field of 0.

	100	200	300	400	500
SMBFT	0.0407	0.0234	0.0169	0.0134	0.0115
Turbo	0.0528	0.0294	0.0166	0.0149	0.0120
SLICO	0.0591	0.0322	0.0207	0.0155	0.0123
ERS	0.0604	0.0319	0.0221	0.0170	0.0138

**Table 5:** Entropy value of image with noise of 5% and bias field of 40%.

	100	200	300	400	500
SMBFT	0.0449	0.0256	0.0186	0.0145	0.0123
Turbo	0.0604	0.0324	0.0222	0.0162	0.0130
SLICO	0.0652	0.0353	0.0226	0.0169	0.0134
ERS	0.0691	0.0364	0.0249	0.0190	0.0154

**Table 6:** Entropy value of segmentation result of 100 superpixel blocks.

	0_00	0_40	5_00	5_40	7_00	7_40
SBMFT	0.0407	0.0407	0.0500	0.0449	0.0470	0.0467
Turbo	0.0528	0.0540	0.0600	0.0604	0.0616	0.0600
SLICO	0.0591	0.0609	0.0647	0.0652	0.0656	0.0656
ERS	0.0604	0.0631	0.0688	0.0691	0.0709	0.0709

**Table 7:** Entropy value of segmentation result of 300 superpixel blocks.

	0_00	0_40	5_00	5_40	7_00	7_40
SBMFT	0.0169	0.0172	0.0189	0.0186	0.0184	0.0185
Turbo	0.0166	0.0206	0.0227	0.0222	0.0229	0.0229
SLICO	0.0207	0.0211	0.0226	0.0226	0.0229	0.0228
ERS	0.0221	0.0227	0.0249	0.0249	0.0255	0.0254

**Table 8:** Entropy value of segmentation result of 500 superpixel blocks.

	0_00	0_40	5_00	5_40	7_00	7_40
SBMFT	0.0115	0.0117	0.0124	0.0123	0.0125	0.0121
Turbo	0.0120	0.0121	0.0132	0.0130	0.0134	0.0133
SLICO	0.0123	0.0125	0.0134	0.0134	0.0135	0.0135
ERS	0.0138	0.0141	0.0154	0.0154	0.0157	0.0158

## Data Availability

Data are available at the Berkeley Segmentation Dataset and Benchmark (http://www.eecs.berkeley.edu/Research/Projects/CS/vision/grouping/segbench/) and Simulated Brain Database (http://brainweb.bic.mni.mcgill.ca/brainweb/).
